# Big Data and Dementia: Charting the Route Ahead for Research, Ethics, and Policy

**DOI:** 10.3389/fmed.2018.00013

**Published:** 2018-02-06

**Authors:** Marcello Ienca, Effy Vayena, Alessandro Blasimme

**Affiliations:** ^1^Health Ethics and Policy Laboratory, Department of Health Sciences and Technology, ETH Zurich, Zurich, Switzerland

**Keywords:** dementia, big data, ethics, health policy, Alzheimer’s disease, real-world evidence, mild cognitive impairment, m-health

## Abstract

Emerging trends in pervasive computing and medical informatics are creating the possibility for large-scale collection, sharing, aggregation and analysis of unprecedented volumes of data, a phenomenon commonly known as big data. In this contribution, we review the existing scientific literature on big data approaches to dementia, as well as commercially available mobile-based applications in this domain. Our analysis suggests that big data approaches to dementia research and care hold promise for improving current preventive and predictive models, casting light on the etiology of the disease, enabling earlier diagnosis, optimizing resource allocation, and delivering more tailored treatments to patients with specific disease trajectories. Such promissory outlook, however, has not materialized yet, and raises a number of technical, scientific, ethical, and regulatory challenges. This paper provides an assessment of these challenges and charts the route ahead for research, ethics, and policy.

## Introduction: Big Data, Health, and Dementia

The predicted threefold increase in the prevalence of dementia in the coming decades (1) will put health-care systems and informal caregivers under an unmanageable pressure. This explains the current eagerness to find a cure, to slow down progression, and to develop better predictive tools to spot early signs of cognitive decline that may develop into full-blown dementias. The identification of biomarkers for Alzheimer’s disease (AD) has paved the way for research into early determinants, with an emphasis on treating patients before the clinical manifestation of the disease (2). According to a study of the drug development pipeline for AD in 2017, current efforts focus mainly on disease-modifying therapies (DMTs) (3), and most of DMT agents in phase III AD trials (as reported on https://clinicaltrials.gov in 2017) address amyloid targets (15 out of 18) (idem). Yet, the failure rate for these kinds of trials is notoriously high (4).

Increased tau protein in the cerebrospinal fluid (CSF) is also used as a biomarker for patients affected by mild cognitive impairment (MCI) who are likely to transition to AD (5). Still, the very use of MCI as a prodromal phase of AD has recently come under attack, as MCI itself reverts to normal cognitive functioning in many patients (6, 7).

Genome wide association studies have identified genes associated with increased risk of late onset AD (idem). In particular, APOe4 has been shown to have some, but only limited predictive value in determining the progression from (MCI) to AD (8).

Further efforts are underway to establish other biomarkers—genetic, biological, and combinations thereof—in both CSF and plasma (2), as well as attempts to look at cognitive decline on longitudinally longer scales (9).

Multidimensional models integrating multiple biomarker data and etiological pathways are regarded as decisively more promising than reductionist approaches based on single explanatory hypothesis like those driving clinical research on the amyloid cascade (4, 10).

Integrative approaches to dementias are ideal candidates to test an incipient approach driven by the use of big data in dementia research. Recently, the global surge of interest around big data has spilled over into the field of dementia, as well as in many other domains of medicine and health research. Big data refers to unprecedentedly large amounts of data analyzed through novel data mining techniques for a variety of different purposes. Although the concept lacks an agreed-upon definition, it is generally assumed that big data is characterized by the so-called 3Vs: volume, velocity, and variety (11). This simplified definition captures some peculiar facts. For starters, the total amount of data stored in data centers in the world is estimated to reach 915 exabytes by 2020 (2.4-fold increase with respect to today) (12). In parallel the amount of data generated by networked end-user devices and appliances will be more than triple in the same period (12). Data are being generated at impressive velocity. Both structured and unstructured data contribute to this rapid growth. Biomedicine does its part too. Sequencing a whole human genome now take as little as few hours, and it is estimated that more than 35 petabytes of genomic data are produced every year (13). But genomic data are not the only reason why health care is predicted to be one of the domains in which big data will have a transformative effect (14). Electronic health records (EHRs)—comprehensive records of patient health history in electronic format—have now entered routine clinical practice in most advanced countries. Sensor-equipped mobile devices, wearables, and appliances keep track of physical activity, location, sleeping habits, and vital parameters in real time, 24 h a day. Mobile-based applications (hereafter apps), loyalty cards, credit cards, and smart objects register accurate data about our consuming habits. Biomedical research produces huge amounts of data that scientists can store and share for secondary use through a variety of data repositories and biobanks. The ability of such heterogeneous information to provide a multidimensional account of one’s health state has led some to speak of it as our “digital phenotype” (15), and to introduce the notion of “digital health” (16). Interest is rapidly growing around the potential medical breakthroughs enabled by mining such unprecedented amounts of data.

Dementia research is among those fields in which big data are regarded as more promising. Leveraging the collection, aggregation, and predictive analysis of large data volumes could reboot dementia research and care as it holds the potential of casting new light on its etiology, enabling timelier diagnosis and prevention strategies, and possibly overcoming current therapeutic limitations. In particular, this potential is more likely to be realized by enabling the integration of EHRs, molecular biomarkers, neuroimaging biomarkers, and mobile health (mHealth) data.

**Figure 1:**
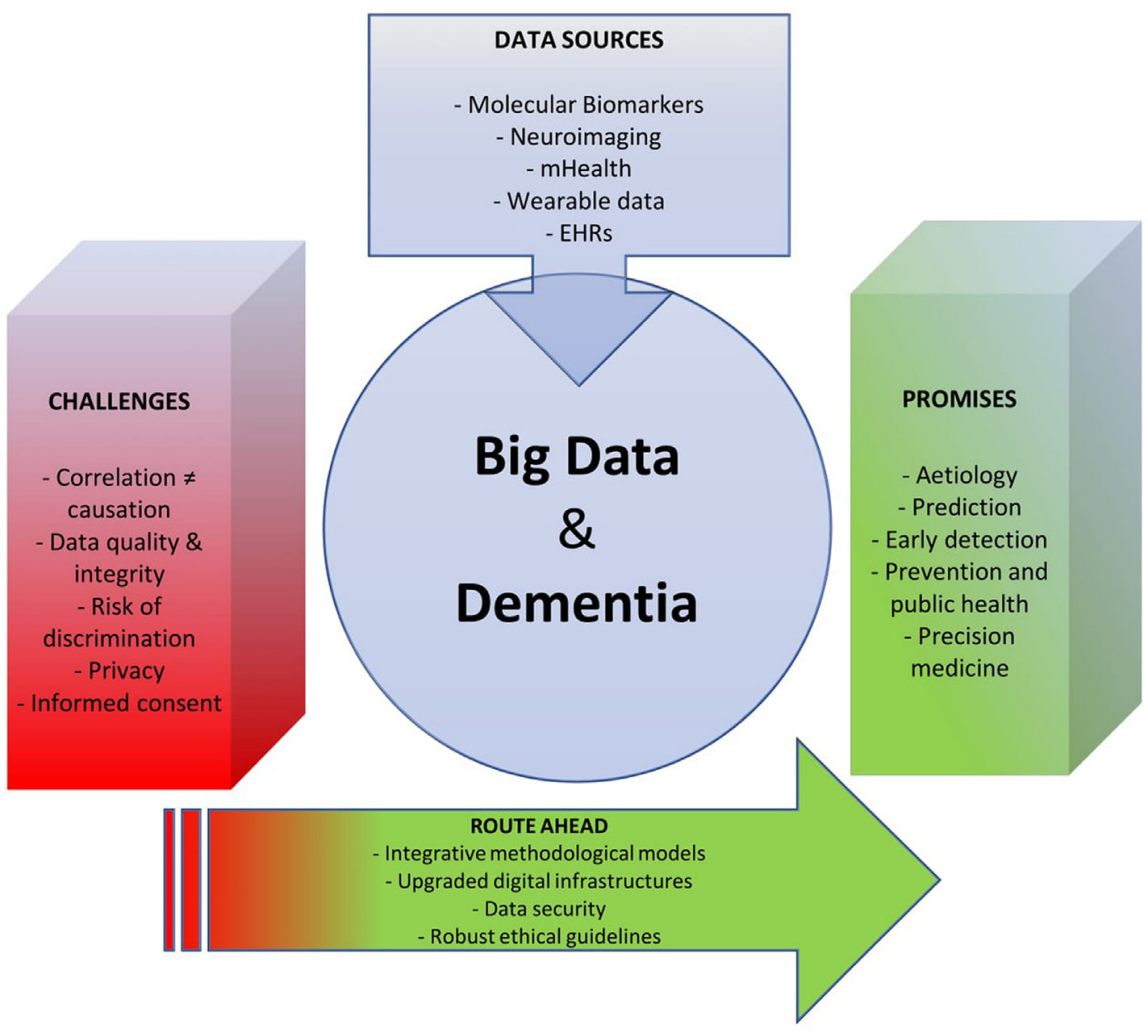
Tackling the global dementia epidemic requires the deployment of all possible scientific and health policy means. Yet, success in this ongoing battle also depends, and in a crucial way, on targeting the right enemy and using available resources efficiently. As the big data turn starts to invest medicine, opportunities emerge to deploy new, effective weapons against this disabling condition. Our analysis revealed that the prospect of using big data for dementia research and care is still in its infancy, and that numerous challenges lie ahead on the way to furthering progress in this field. An integrative approach can overcome such obstacles and help reap the benefits of big data in dementia research and care.

In this contribution, we review the existing scientific literature on big data approaches to dementia including both original research articles and commercially available mHealth applications. Based on this synthesis, we identify some major promises and challenges associated with big data trends in dementia research and care and chart the route ahead for research, ethics, and policy (see Figure 1).

## Methods

In spite of their disruptive potential, big data trends in dementia still remain a relatively unexplored topic. In October 2017, we conducted a PubMed search [(“big data” AND “dementia”) OR (“big data” AND “cognitive decline”)] with unrestricted time range. The search retrieved nine peer-reviewed articles and one conference paper. The nine scientific articles included editorials (17, 18) and commentaries aimed at introducing big data principles to the dementia community, examining the applicability of these principles to dementia research or discussing the level of preparedness of the dementia community for big data approaches (19). While most articles focused primarily on AD dementia, a recent commentary explored the use of big data resources to optimize data use in vascular dementia research (20).

The data sources examined in these studies included EHRs (20), Internet searches (21), and genetic data (22). Additional data sources such as mHealth data did not appear in the foreground of current scientific literature. However, a parallel search on the two main digital distribution services (app stores), namely, Google Play and iTunes (the latter screened through API search), indicated the current availability of 35 mobile apps aimed at screening dementia and cognitive decline through self-assessment tools or digital assistants for health professionals.

Further screening was performed through unstructured online search and retrieval of secondary sources. This second phase of analysis revealed that, besides traditional research models, big data approaches to dementia have also been pursued in the form of data analytics challenges. For instance, the DREAM Challenge for AD aimed at: (i) predicting changes in cognitive scores 2 years after initial assessment; (ii) understanding the biological basis of resilience to amyloid pathology; and (iii) classifying individuals into diagnostic groups using neuroimaging. The challenge capitalizes on large datasets such as the Alzheimer’s Disease Neuroimaging Initiative’s database and leverages multiple data sources including genetics, neuroimaging, cognitive assessment, and demographic information.

While research articles on big data and dementia still appear relatively rare, several policy documents produced by intergovernmental organizations focus on this topic. Between 2013 and 2015, the Organisation for Economic Co-operation and Development (OECD) released five policy papers addressing promises and challenges of big data trends in dementia care and research (23–27).

## Results

### Promises

As often observed, dementia is “both a global problem and a pathological conundrum” (18). Therefore, the deployment of big data techniques should aim both at alleviating the global burden of dementia and at providing novel explanatory resources for the further understanding of the disease.

Big data may in the future help in cracking the pathological conundrum of AD by shedding new light on its etiology. The use of single molecular biomarkers in isolation has so far not successfully predicted the functional and cognitive outcomes of dementia (28). Based on this observation, researchers have criticized the disproportionate focus on single molecular biomarkers such as amyloid-β and tau in dementia research, and called for more integrative approaches to the study of the etiology (10, 29). For this reason, big data trends could corroborate a multi-modal (having different modes of data acquisition from heterogeneous data sources) and multi-scalar (from the molecular to the behavioral and population scale) account of dementia, enabling statistical associations across different data types and scales. This might fix the lack of integration that currently affects different data levels, hence helping to “glimpse the big picture” (30)—from genes and molecules to cognition and behavior—in dementia research.

In recent years, attempts to integrate and statistically correlate information across large population groups have been used to achieve earlier detection of AD, ideally in the pre-symptomatic phase. Researchers have utilized multiple data sources including neuroimaging (31, 32), hand gesture tracking (33), and retinal scans (34), and developed machine learning algorithms to detect structural (e.g., changes in brain structure) or functional (e.g., changes in time response during the completion of tasks) anomalies years prior to the onset of symptoms. Some of these studies show prognostic and predictive potential (32, 33). In parallel, a number of smartphone-based apps claim the ability to detect early signs of cognitive decline through gaming behavior or digitalized mental examination. For example, the mobile app *Sea Hero Quest* (35) gamified virtual spatial navigation assessment techniques (36) to find correlations between levels of gaming performance and cognitive decline (37). With over 2.5 million users, the app can capitalize on larger datasets than most conventional clinical tools, hence leveraging the potential of big data analytics to improve screening and early detection. While Sea Hero Quest is designed for self-assessment, other apps such as *CogniSense* (38) and *ACEmobile* (39) provide digital assistance to clinicians during the cognitive screening of patients.

Employing big data to reduce the global burden of dementia (25) implies aggregating large sets of population-scale data to improve prevention and public health strategies. For example, Doubal et al. have argued that the linkage of routine clinical data at a national or even international level might result in a better understanding of the risk factors of dementia, resulting in more effective prevention (20). In a similar fashion, Dacks et al. have argued that optimizing the use of observational data through data-pooling and Internet-based tools might support personal, clinical, or public health decision-making and contribute to the development of specific interventions that reduce modifiable risk factors (40). In parallel, as stated in an OECD report, integrating data from different size scales (from molecular to whole population) and information types (from neurophysiological to behavioral) might enable the development of precision medicine solutions (24). With recent unmet expectations regarding pharmacological therapy, this *personalized turn* can increase therapeutic effectiveness through the customization of treatments and medical decisions to each individual patient (41, 42). This is particularly relevant as evidence suggests that the “biological processes driving the clinical phenotype can differ remarkedly from patient to patient” (28) and can also depend on comorbidities, co-medications, and patient genotypes.

### Challenges

Big data and digital health tools can streamline the detection of early signs of cognitive decline. MCI is a prominent example of a prodromal syndrome that, while not strongly debilitating, can develop into full-blown dementia (43).

Other entities have recently been suggested to have a similar predictive value, such as subjective cognitive decline and mild behavioral impairment (44, 45). Apps that monitor the clinical presentation of such conditions (such as users’ mood and cognitive performance over time) can greatly facilitate spotting changes from baseline functioning levels. The ubiquity and ease-of-use of such tools may enable self-monitoring practices, thus leading to a widespread increase in the number of patients referred to neurologists. On the one hand, if this enhances early detection of patients at risk of developing dementia, such practices can inflate the number of false positive diagnoses. This is a concrete risk, especially in light of recent findings showing that conditions like MCI, for instance, are prone to stabilization or even spontaneous reversion to normal functioning (46). In the absence of adequate evidence and guidelines, excessive emphasis on preclinical syndromes can lead to over-diagnosis and unnecessary medicalization, with consequences for both health systems and patients. The latter face risks of insurance and employment discrimination, stigmatization, and direct psychological harm. Health systems, on the other hand, may have to cope with unnecessary financial and organizational costs in response to an upsurge of mild/preclinical syndromes (46–48).

These downstream effects of mHealth applications in dementia demonstrate the need to establish *ad hoc* regulatory pathways to validate apps that make medical claims. Regulators such as the US Food and Drugs Administration (49) and the UK Medicines and Healthcare Products Regulatory Agency (50) are starting to venture in this domain. Nonetheless, we observed that many developers present their applications in ambiguous ways, without offering sufficient information regarding either the evidence backing their products, or the way in which they will handle the personal data of their customers. More effective incentives and disincentives are still needed to ensure sufficient consumer protection in this area. In particular, there needs to be more stringent oversight regarding the health-related claims of apps.

Despite these drawbacks, early detection remains laudable, as it allows us to treat patients before degenerative trajectories compromise the odds of slowing or stabilizing cognitive decline. The collection and analysis of multiple data types describing the aging trajectory of individual patients can help isolate discrete stressors and molecular characteristics (including genetic ones), and cluster them with cognitive or neuro-psychiatric symptoms that lead to the clinical manifestation of dementia. This kind of knowledge will improve the clinical understanding of dementia, as well as the development of personalized therapeutic and preventive interventions. Creating the evidence base for such interventions will require large-scale personal data repositories, giving rise to regulatory challenges in terms of data management, protection, aggregation, interoperability, privacy, and informed consent for the collection, use, and sharing of such data. Such issues are currently being debated in ethics and regulatory circles (51). Yet, other pressing issues relate to the most adequate means to generate clinically reliable and usable evidence from heterogeneous “real-world data,” such as EHRs, mobile device data, and socioeconomic data (52). Current discussions on pragmatic trial designs will likely turn out relevant to research in novel, data-driven approaches to prevention and therapy in dementia (53). In the case of a stigmatizing condition like dementia linking data from within and without the clinical setting to detect early signs of cognitive decline poses peculiar ethical challenges. At a minimum, patients (or their legal representatives, in case of advanced dementia) should be made aware of such activities and be given the option to opt out.

## A Way Forward: The Need for an Integrative Approach

### Scientific Evidence and Theory

To overcome the above structural challenges and ensure the success of big data initiatives for dementia, there is a need for integrative approaches at the level of research methodology, digital infrastructures, and financing, as well as ethics and regulation.

At the scientific level, there is a need for clearly demarcating the explanatory power of big data driven research. Large-scale data collection and further mining through analytical tools could boost dementia research and care management, establishing reliable statistical correlations between heterogeneous data sources whose association could not be detected through small-scale methods. It is questionable, however, whether big data approaches alone would suffice to uncover the causal mechanisms of AD pathology. The idea that large datasets might *speak for themselves* (54), independent of explanatory hypotheses (55) has attracted praise as well as criticism (56, 57). Integrative approaches are needed to combine the predictive power of big data with theoretically robust and causally explanatory scientific models. A valid proposal for such integration has been advanced by Geerts et al., who suggested that data-driven analytic approaches in AD research “need to be organically integrated into a quantitative understanding of the pathology” involving mechanism-based modeling and simulation approaches. In their view, this integration could enable a shift from big data to *smart-data*, i.e., from “information” to “actionable knowledge” (28). Similarly, DeKosky has called for the integration of big data approaches with basic neuroscience (18).

Integrative and theory-mediated big data approaches are well placed to overcome current limitations in dementia research. Taking a stance from systems biology and complexity theory, Geerts et al. argued that the integration of big data analytics with modeling and simulation might overcome the explanatory failures of reductionist biological approaches focused on single molecular biomarkers in isolation. In their view, this holistic approach has already shown benefits in PD-dementia, as it has led to a better understanding and optimization of deep-brain stimulation protocols (28).

### Digital Infrastructure

Current digital infrastructures of dementia research and care need to be upgraded. As stated in a recent OECD report, secure infrastructures for data storage, processing, and access need to be sustained through complementary resources (27). While ongoing digitalization and automation in dementia care offer novel opportunities for large-scale data acquisition (58), further efforts are required to sustain the secure and reliable sharing of such information, and to guarantee the interoperability of different data-repositories (e.g., genetic biobanks, neuroimaging repositories, and eHealth data platforms). Active cooperation between public and private actors has been recognized as a viable strategy for increasing funding opportunities and favoring the digital transformation of dementia research and care (26). Yet, the appropriation of health data by large ICT corporation can cause public unease, thus undermining the development of data-driven medicine. A recent controversy over Google’s access to NHS data through its AI subsidiary DeepMind shows that sufficient safeguards are not yet in place (59).

### Ethical Guidelines

Ethically robust guidelines for the collection and sharing of personal health data would facilitate big data research while maintaining public trust and protecting data subjects. Existing oversight mechanisms (such as ethics review) and conventional informed consent models appear “ill suited” for large-scale data collections (60, 61). As far as research on large-scale repositories of structured and unstructured data is concerned, *ad hoc* criteria for assessing research protocols employing novel data analytics tools are urgently needed. Moreover, informed consent—in its current shape—does not grant data subjects (nor their legal representatives) sufficient control over highly sensible information regarding their cognitive state. This is a disincentive for people to make their data available for research in the first place. New mechanisms are being explored to enable more granular data control on the part of data subjects—for example by implementing data portability rights, or by introducing electronic consent management mechanisms and participatory forms of data governance (61). The longitudinal nature of studies based on big data and real-word evidence, however, poses issues relative to the validity of initial consent obtained from people whose cognitive functions may degrade over time. *Ad hoc* oversight mechanisms, such as advanced directives, shall be in place to safeguard the autonomy and wellbeing of those patients while at the same time enabling scientific progress.

### Privacy and Data Security

As to privacy and data security, researchers and regulators need to acknowledge that not even anonymization of deep phenotypic data—such as those that are needed for the development of big data approaches in dementia—is a sufficient firewall. As demonstrated in the case of genomic (62) and electrophysiological data (63), maliciously re-identifying anonymous data is within reach of sufficiently skilled offenders. Given the sensitive nature of data regarding cognition (64) and given the frequency of spectacular health data breaches (65), data security represents a priority. It follows that efficient regulatory and technical measures to shore up data security are key to scientific progress in the field. Privacy-preserving techniques such as encryption and block chain need to be incorporated into the digital infrastructure of current data transmissions. These solutions will not only enhance data security but also facilitate and sustain the trust of individuals (both healthy subjects and people with symptomatic dementia) in the data collection and sharing (66).

## Author Contributions

All authors listed have made a substantial, direct, and intellectual contribution to the work and approved it for publication.

## Conflict of Interest Statement

The authors declare that the research was conducted in the absence of any commercial or financial relationships that could be construed as a potential conflict of interest.
